# A Successful Record

**Published:** 1889-06-08

**Authors:** 


### The Hospital

A Weekly Institutional Journal of

Science, Medicine, Nursing, and Philanthropy.

*For Hospitals, Asylums, Medical Practitioners, Students, Nurses, Families, Parishes, Congregations, and the Charitable Public.*

Vol. VI. No. 141.] [June 8, 1889.

### A Successful Record.

Public bodies, whether of the voluntary kind or legally constituted, never succeed in winning every man's approval. Nevertheless, in this country they almost invariably survive and grow if the heart of their purposes and aims be sound and wholesome. The average Briton has a fund of homely sense, and a certain doggedness of resolution which make him stick to a thing he has once put his hand to. If in spite of all the fiery criticism which may be let loose upon him from without, and all the defections of faint hearts and time-servers from within, he still believes in his cause, then, like his ancestors of Crecy and Poictiers, of Magna Charta and the banishment of the Stuarts, he sturdily buckles on his armour and stands or falls by his choice. That is surely the right way as well as the British way! How can any good work ever be got forward if those who take it in hand are for ever starting from the road, like jibbing horses, to the imminent risk of their riders' limbs or necks?

The only thing that The Hospitals Association needed eighteen months ago was some kind of whip or spur to make it practical. It had talked for several years to little purpose. Then came the split, the defection, the opposition; and the Association felt that the time had come when it must "do or die." In the course of the last eighteen months it has accomplished more with less means than, perhaps, any other voluntary association in the metropolis or the country. What other body consisting of two or three hundred members, and with no subscription list, can show such striking and valuable practical results as the National Pension Fund for Nurses, and the establishment of a complete scheme of Street Ambulance for the largest city in the world? All this has been done without flourish of trumpets or fuss of any kind, and under the presidency of one of the most unassuming though one of the most accomplished and dignified living representatives of British medicine. It is a curious irony of fate which sometimes compels the most peace-loving men to lead the stormiest battles. If any man in the world hates war and fuss and public notoriety it is Dr. Bristowe. Yet when no other man of eminence in the medical profession was prepared to march at the head of what appeared to many to be a forlorn hope, without hesitation he responded to the call to leadership, and his leadership has led to victory, honour, and splendid public service all along the line. No dog wags his insolent tongue or points the finger of scorn against The Hospitals Association to-day. Its work is known to the whole metropolis, and that work is of such a kind that all critics are silent, and all fair-minded men and women generously approve.

It is impossible to exaggerate the importance of the National Pension Fund for Nurses, as it is impossible to overestimate the value of trained nurses to the public at large. Trained nursing has revolutionised practical medical treatment. It has reduced it, if not to an exact science, at least to a highly scientific art. For the performance of such duties as the physician and the public commit to the trained nurse, there is required a high degree of specially-instructed intelligence and great trustworthiness of personal character. Hitherto it has not been difficult to obtain trained nurses in adequate numbers, because the public honour and distinction gained by a few eminent names have made sick nursing popular to the impressionable female mind. But nursing, as a profession, is yet in its infancy. The first generation of nurses are still in full work. We have not yet a whole generation who have passed their working days and commenced the effort to live in old age without stored resources. If we had had, not one, nor two, but several generations of trained nurses who had found out by practical experience that sick nursing means hard, devoted, and exhausting labour in early and mid-life, but starvation and neglect in old age, it is quite certain that the supply of candidates for this noble profession would have been very much smaller than it is to-day.

It is here where the Pension Fund is so invaluable. It comes in at the very point where it is needed to save a meritorious class from poverty in old age, and the public who have used the services of that class will now be able to protect it from the humiliation and shame which such poverty would have brought to every right-thinking mind. Neither nurses nor the public will ever know the real value of the Pension Fund, because they will never see in actual experience thousands of aged and helpless nurses, after lives of highly-intelligent and self-denying work, reduced to the level of distress and want. Trained nurses are now occupying, and are destined to occupy, a sphere which is similar in character to that occupied by the clergyman, the schoolmaster, and the doctor. Who could contemplate without deep shame the spectacle of the whole body of clergymen or medical men reduced to pauperism and chance charity, or the workhouse, when their active days were past? Still less, if that were possible, could we bear for a single moment to think of women, whose whole days have been shut off from the ordinary prospects and pleasures of woman's life, being discharged from the army of useful workers in old age, and, like worn-out horses or toothless dogs, left to the tender mercies of cold hearts and unfeeling minds. The Pension Fund renders all that impossible; and its inevitable effect must be to raise and maintain at a constantly heightening level the whole nursing profession, and thus to preserve to the public an unfailing supply of those trained nurses who, in the judgment of the most eminent members of the medical profession, are as necessary in all cases of serious illness as the medical man himself.

The ambulancing of every district of London so that no man who falls down or is injured in any street in the metropolis, whether central or remote, need be more than five minutes before he is on his way to the hospital lying on a comfortable ambulance litter, makes a more direct appeal to public imagination than even the National Pension Fund. To our thinking, it is less important in itself and less wide-reaching in its effects. But it was a work which urgently demanded to be done, which everybody desired to see done, and yet which nobody knew exactly how to set about doing. Until Mr. Thomas Ryan worked out the subject, the most conflicting opinions prevailed both as to what was needed and the probable cost of the work. It is much to Mr. Ryan's honour that in the course of a very few weeks he mastered all the facts and difficulties of the question, and produced the neucleus of a scheme which for economy and efficiency could not possibly be surpassed.

The scheme so commended itself to a few wealthy philanthropists, that they at once placed the whole sum (£1,500) for the first outlay at Mr. Burdett's disposal, and Mr. H. L. Bischoffscheim became treasurer to the movement. Thanks to the initiative of The Hospitals Association, London will shortly be furnished with as complete and well-equipped a system of Street Ambulance as can possibly be desired.

We have left ourselves no space for the consideration of several important papers which were read and discussed at the various sessional meetings of the association held at St. Bartholomew's, Guy's, St. Thomas's, and other hospitals. In addressing the members at the closing meeting at the Westminster Town Hall on Wednesday, Dr. Bristowe called special attention to some of those papers, notably that on "Hospital Extravagance," by Mr. Michelli, and that on the "Pay System," by Dr. Gilbart Smith. The President affirmed that his long experience of hospitals convinced him that there was solid ground for many of Mr. Michelli's most serious charges. No one will accuse Dr. Bristowe of being either an alarmist or a fire-eating reformer. If he admits, as he does, that Mr. Michelli's paper was, on the whole, a faithful epitome of facts, few who know him will doubt that there is something fundamentally wrong in a good deal of the hospital management of the times. This The Hospitals Association, which consists so largely of hospital managers, is bound to enquire into. If, at the close of the coming winter session, it can give as good an account of its work in dealing with the urgent question of hospital extravagance as it did when it took up the National Pension Fund and the Ambulance questions, it will have earned a still stronger and more enduring title to the gratitude of the public than it now enjoys. The public, on its part, will do well to give the Association all the moral and material assistance which it requires, and so well deserves.

